# Implication of Heat Shock Factors in Tumorigenesis: Therapeutical Potential

**DOI:** 10.3390/cancers3011158

**Published:** 2011-03-07

**Authors:** Aurelie de Thonel, Valerie Mezger, Carmen Garrido

**Affiliations:** 1 INSERM U866, Dijon, France; E-Mail: aurelie.dethonel@u-bourgogne.fr (A.D.T.); 2 Faculty of Medicine and Pharmacy, University of Burgundy, 21033 Dijon, France; 3 CNRS, UMR7216 Epigenetics and Cell Fate, Paris, France; 4 University Paris Diderot, 75013 Paris, France; 5 CHU, Dijon BP1542, Dijon, France

**Keywords:** Heat Shock Factors, cancer, therapeutical approaches

## Abstract

Heat Shock Factors (HSF) form a family of transcription factors (four in mammals) which were named according to the discovery of their activation by a heat shock. HSFs trigger the expression of genes encoding Heat Shock Proteins (HSPs) that function as molecular chaperones, contributing to establish a cytoprotective state to various proteotoxic stresses and in pathological conditions. Increasing evidence indicates that this ancient transcriptional protective program acts genome-widely and performs unexpected functions in the absence of experimentally defined stress. Indeed, HSFs are able to re-shape cellular pathways controlling longevity, growth, metabolism and development. The most well studied HSF, HSF1, has been found at elevated levels in tumors with high metastatic potential and is associated with poor prognosis. This is partly explained by the above-mentioned cytoprotective (HSP-dependent) function that may enable cancer cells to adapt to the initial oncogenic stress and to support malignant transformation. Nevertheless, HSF1 operates as major multifaceted enhancers of tumorigenesis through, not only the induction of classical heat shock genes, but also of “non-classical” targets. Indeed, in cancer cells, HSF1 regulates genes involved in core cellular functions including proliferation, survival, migration, protein synthesis, signal transduction, and glucose metabolism, making HSF1 a very attractive target in cancer therapy. In this review, we describe the different physiological roles of HSFs as well as the recent discoveries in term of non-cogenic potential of these HSFs, more specifically associated to the activation of “non-classical” HSF target genes. We also present an update on the compounds with potent HSF1-modulating activity of potential interest as anti-cancer therapeutic agents.

## 1. Introduction

The cellular response to proteotoxic stress, historically called “Heat Shock Response” (HSR), is strongly conserved through evolution and involves the activation of transcriptional regulators named Heat Shock Factors (HSFs). The mammalian HSF family consists of four members (HSF1, HSF2, HSF3, and HSF4) and was first characterized as master transcription factors by their abilities to regulate the expression of a set of highly conserved proteins, called Heat Shock Proteins (HSPs). HSPs function mainly as molecular chaperones and display strong cytoprotective effects against stress-induced proteotoxic damage by preventing protein aggregation, targeting misfolded proteins for degradation, or blocking the apoptotic pathway. Beyond the regulation of *Hsp* genes, HSFs have been involved in the regulation of numerous other genes activated by heat and other stresses [1–4]. In addition, under non stressful conditions, HSFs target a wide spectrum of genes involved in a variety of biological processes including cell maintenance, differentiation and development [2–6]. Last but not least, HSF1 is a conserved regulator of aging by promoting longevity, and has been involved in age-related neurodegeneration (Reviewed in [7–9]).

The universally conserved abilities of HSFs to promote cellular adaptation and survival in response to environmental stress are accompanied by a Janus-like behavior, which can favor oncogenic transformation [10]. The first link between HSR and cancer was provided by the observation that aberrant expression of HSPs is frequently associated with cancer. But, the role of HSF1 in cancer far exceeds the sole regulation of *Hsp* genes since there is not always a correlation between the high HSF activation and/or expression levels frequently found in cancer cells, and those of HSPs [11–13]. In fact, there is growing evidence that the role of HSFs in tumorigenesis involves the induction of, at least some, classical heat shock genes, as well as many “non-classical” targets. In this review, we present recent discoveries in terms of oncogenic potential of HSFs, and notably those associated with the activation of “non classical” HSF target genes.

## 2. HSF Structure, Expression and Function

### 2.1. Structure/Activity

**Figure 1.**
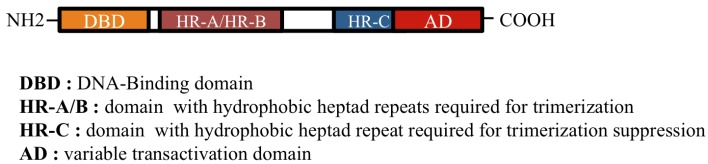
Structure of Heat Shock Factors (HSFs).

HSF family members (HSF1, HSF2, HSF3, and HSF4) display unique as well as overlapping functions, show tissue-specific patterns of expression, are submitted to numerous post-translational modifications, and interact with many protein partners [14–18]. HSF proteins are composed of functional domains, among which the most conserved is the amino terminal DNA-binding domain (DBD). Upon heat shock, thanks to its oligomerization domain (heptad repeats HR-A/B), adjacent to the DBD, the latent inactive monomeric HSF1 forms a trimer and is subjected to extensive post-translational modifications. In normal conditions, this assembling is prevented by another heptad repeat domain HR-C [19,20], which specifically interacts with HR-A/B domains and maintains HSF1 in a monomeric conformation (Figure 1). The deletion of the HR-C domain results in constitutive trimerization which may explain why HSF4, that lacks this domain, has a constitutive DNA-binding ability [19]. HSF2 and HSF3 are present as a dimer [21] and share the ability to bind DNA sequences, called Heat Shock Elements (HSE), originally defined as succession of several inverted repeats of the pentanucleotide motif NGAAN and recently refined by genome-wide analysis [3]. HSF family members exhibit different binding site preferences in terms of architecture and composition of the HSE, thereby allowing a great diversity in the regulation of specific target genes [4,22,23]. In this respect, HSFs are able to act as both activators and repressors depending on the environmental conditions and the targeting genes [1–3]. In addition, subtle crosstalks exist between HSFs, which, together with the combinatorial possibilities allowed by the different post-translational modifications, multiply the levels of fine-tuning the expression of HSF target genes. Furthermore, in specific conditions, HSF1 and HSF2 can form heterotrimers through which HSF2 transiently modulates the very potent and dominant HSF1 activity on its target genes [24–28].

Upon stress, HSF1 and HSF4 are able to recruit chromatin remodelers SWI/SNF [29,30] and to target stress-specific histone modifications [31]. In human cells, HSF1 was shown to direct the transcription of satellite III DNA in pericentromeric heterochromatic regions of particular human chromosomes that are strikingly characterized by an epigenetical status euchromatin. Therefore, stress-activated HSF1 can trigger the conversion of a partially constitutive heterochromatin status to a transcription competent status [32–34]. Likewise, HSF2 has an epigenetic role, strictly speaking, in the heritability of a decondensed chromatin status on the *Hsp70* gene. During mitosis, HSF2 inhibits chromatin condensation through interaction with condensin, in a process called ≪bookmarking≫ [35].

### 2.2. Expression and Physiological Functions

Besides their role in stress response, HSFs assume specific and non-overlapping functions which enlighten their role in cancer. They regulate proliferation, asymmetric division, differentiation, migration, and survival by either activating or repressing their target genes (Table 1).

- **HSF1.** The ubiquitously expressed HSF1, known as a master controller of the cellular-response to different stress conditions, is a maternal factor, stored in the oocyte, and essential for oogenesis and preimplantation development through the regulation of *Hsp90*α gene expression [36–38]; reviewed in [5]. It is also required for the development and maintenance of tissues including the adult brain [39–41] germ cells [42–44], ciliated cells [45], and immune cells [46,47], regulating both *Hsp* and non-Hs*p* target genes.
- **HSF2.** As mentioned above, HSF2 is able to finely tune the HSR mediated by HSF1. However, its role in stress is more striking upon proteasome inhibition, because it regulates the expression of proteasome subunits [6]. In the context of differentiation, as in stress, a pool of nuclear HSF2 can associate with HSF1 and modulate the expression of heat-shock proteins [24,26] as well as noncoding satellite III RNA in nuclear stress bodies (unique subnuclear organelles which form in response to heat shock) [27]. Interestingly, HSF2 is mainly associated with brain development and gametogenesis [48–50] where it displays a spatiotemporal expression pattern [5,51]. In the developing brain cortex, HSF2 regulates multiple aspects of neuronal migration through gene expression, like p35, a crucial activator of the serine/threonine kinase, Cdk5. In spermatogenesis, HSF2 cell-specific expression is negatively regulated by microRNA miR18, which belongs to the Oncomir-1 cluster associated with tumorigenesis [52]. HSF2 and HSF1 can form heterotrimers in testis [27] and share common target genes during spermatogenesis, in particular sex chromosomal multicopy genes [15,53]. HSF2 binding to its target genes during spermatogenesis is associated with histone H4 acetylation [53]. The inactivation of both HSF1 and HSF2 leads to completely male sterility, reinforcing their intertwined functions, which also rely on global chromatin remodeling.
- **HSF3.** Initially only identified in the chicken (cHSF3), this ubiquitously expressed factor has been recently identified in the mouse (mHSF3). In human, HSF3 might be present as a pseudogene, since no transcripts have been detected [54]. In contrast to others HSFs, HSF3 does not display the same regulatory role in different species [55]. Whereas cHSF3 is the major HSF in response to heat shock and therefore the main inducer of HSPs, mHSF3 does not induce classical *hsp* genes, but instead induces the expression of other stress-responsive genes, raising the possibility that it could regulate distinct sets of genes in development or longevity [18].
- **HSF4.** Mainly expressed in the human heart, brain, skeletal muscle, and pancreas [56], HSF4 has been shown to be required for the development and maintenance of sensory organs [5,57,58]. During this process, HSF4 either cooperates or competes with HSF1 for common target genes, including members of the Fibroblast Growth Factor (FGF) family, in a cell-specific manner in lens and olfactory epithelium [45,59]. Recently, it has been reported that HSF4 binds to more flexible consensus HSE, which are not found in the promoter proximal regions of *Hsp* genes, but are preferentially located in introns, exons and distal regions. HSF4 binding is associated with reduced histone H3K9 methylation. Among the different targets of HSF4 after a heat shock, 33% are non-classical heat-shock genes [4]. Substantial proportions of these regions are also bound by HSF1, reinforcing the concept of their mutual interactions.

Overall, not only HSF1, but also other HSF family members, play significant roles in the induction of non-classical genes in response to heat shock or in developmental conditions. In this respect, HSFs regulate new target genes which include proteostasis genes (proteasome subunits), growth factor genes (FGF, Leukemia Inhibitory Factor (LIF)), and genes that are directly or indirectly involved in cytoskeleton dynamics (*Hsp90* and cortical actin in eggs [37,38]; *p35/p39/Cdk5* [50]; *b_IV_* *tubulin* in ciliary beating activity [60]; *Bfsp*, lens specific intermediate filaments [61]). HSFs also direct the establishment of epigenetic marks and might impact genome structure, *i.e.* chromatin condensation state in spermatogenesis; histone methylation or acetylation status and likely retrotransposons [4,53,62].

## 3. HSFs in Cancers

### 3.1. Multiple Roles of HSF1 in Cancer

#### 3.1.1. Role of HSF1 in the Initiation and Maintenance of Transformed Phenotypes

HSF1 has been involved in the etiology of cancer by its multiple effects in facilitating transformation and tumor invasiveness in response to diverse oncogenic stimuli [10]. In this respect, *Hsf1*^−/−^ mice show lower incidence of tumors induced by mutations of the RAS oncogene or a hot spot mutation in the tumor suppressor p53, and they show improved survival [10]. HSF1 is also crucial for cell transformation and tumorigenesis induced by the human epidermal growth factor receptor-2 (HER2), an oncogene responsible for breast tumors aggressiveness. Knockdown of HSF1 leads to growth arrest and senescence of HER-2-expressing cells [63]. Moreover, HSF1 is required for the maintenance of the transformed phenotype in: (i) established oncogenic cell lines; (ii) breast cells lines with progressive oncogenic states (*i.e.*, primary human mammary epithelial (PHME) cells; immortalized human mammary epithelial (HME) cells; fully transformed and tumorigenic HME cells (HMLER); [64]); (iii) a collection of breast cell lines derived from spontaneous human tumors, with various p53 status (MCF-7 or various mutant alleles BT-474, MDAMB-231, and T47D); (iv) malignant cells of diverse histological origins either derived from human tumors (HeLa (cervix), PC-3 (prostate), and S462 and 90-8 (peripheral nerve sheath) or derived by *in vitro* transformation (293T; kidney). Furthermore, in immortalized MEFs, HSF1 is essential for basal and EGF-induced migration, a process crucial for tumor invasion and metastasis [65].

HSF1 is often elevated in human cancer cells compare to normal cells. However, somatic mutations in *Hsf1* have not yet been identified in human cancers, and overexpression of HSF1 does not lead to transformation, as mutant RAS does. Here, HSF1 does not display typical oncogenic features *per se* but represents a mechanistic and therapeutic challenging target. It is possible that the core function pathways regulated by HSF1 in normal cells might be massively mobilized in cancer cells, like signal transduction, ribosome biogenesis, translation and glucose metabolism [10]. Indeed, cancer cells growth and development highly depend on normal cellular functions governed by genes, which are not typical cancer related genes, and through which HSF1 can promote tumorigenesis.

The unique HSF present in fission yeast cells is essential for normal growth and drives the transcription of target genes that encode proteins with a broad range of biological functions, including protein folding and degradation, energy generation, protein trafficking, maintenance of cell integrity, small molecules transport, cell signaling and transcription [66,67]. Conversely, in murine cells, HSF1 is more dispensable for cell growth and survival, although *Hsf1*^−/−^ mice show defects in postnatal growth and placenta formation, suggesting that in normal cells HSF1, while a major actor in the heat shock response, is also important for other cellular process [68].

In normal mammalian cells, HSF1 is known to regulate *Hsp* genes, but also a wide range of non-*Hsp* genes upon stress or differentiation [2,3]. In aggressive cancer cells, HSF1 is expressed at high levels, which could amplify its activity and broaden the spectrum of its targets. By analogy to what happens in yeast, HSF1 could regulate central functions of the cell biology, in addition to the already known HSP expression. However, although the levels of HSPs (especially HSP27, HSP70 and HSP90) are elevated in different types of cancers, there is not always a correlation between HSF1 constitutive activation and HSP expression. In this way, genetic knockdown of HSF1 fails to induce a decrease in the levels of HSPs in some cancer cell lines [69]. It is believed that the elevated expression of HSF1 in cancer cells does not lead to a kind of global “heat shock response”, but only specific members of the HSP family are induced in a tumor-dependent manner. That explains why HSP expression in cancer cells displays considerable variation, depending on the HSP member and the tumor characteristics. For instance, in HER-2 (human epidermal growth factor receptor-2 oncogene) expressing cells, in aggressive breast cancer models, HSF1 knockdown is accompanied by a specific down-regulation of HSP27 and HSP70 expression [63].

#### 3.1.2. HSF1 and Prostate Cancers

HSF1 expression has been shown to be elevated in prostate carcinoma compared to its normal counterpart [70]. An elevated protein expression of HSF1 and some HSPs was reported in the aggressively malignant cell lines DU145 and CA-HPV-10. However, no difference in the RNA level was detected suggesting that HSP induction was not mediated by HSF1-dependent transcription. In addition, growth of PC3 cells *in vivo*, as tumor xenografts, was accompanied by a marked decrease in HSPs expression (HSP27, HSP70, HSP60, HSP90) whereas HSF level was not modulated. In a PC3 metastatic variant (PC3M), a strong increase in HSF1 mRNA was observed, as well as an increase in HSF1 protein and nuclear localization [71]. Overexpression of HSF1 in non-metastatic PC-3 cells was accompanied by an upregulation of HSP27 at the protein level, but HSP70 and HSP90 were not affected, once again pointing out that the elevated expression of HSPs in cancer cells does not always depend on HSF1. Interestingly, in prostate cancer tissue, HSP27 upregulation, but not HSP70 or HSP90, was significantly associated with clinicopathological factors [72]. In prostate cancer cells, HSF1 also influences the development of an aneuploid state and mitotic progression [73].

#### 3.1.3. HSF1 and Breast Cancers

HSF1 may partly participate in breast cancer progression by inducing specific HSPs, mainly HSP27 [74]. In breast cancer, the highly malignant factor heregulin beta-1 (HRGβ1) is a secreted factor, which binds to c-ErbB-3 and -4 receptors and provokes the recruitment of c-erbB-2 and receptors heterodimerization. The binding of HRGβ1 to the cell surface induces an increase in HSF1 levels that results in anchorage-independent growth and protection from cisplatin-induced apoptosis mediated by HRGβ1 [74]. One aspect of this process relies on the inhibition of GSK3, a kinase that antagonized HSF1 activation. Part of the anti-apoptotic effects of HSF1 seems to operate *via* activation of the *Hsp*70 promoter and therefore likely involves HSPs. Remarkably a crosstalk between the beta-catenin/Wnt pathway and the heat shock cascade has been identified in breast cancer tumors with high metastatic potential. Indeed, the formation of a complex between beta-catenin, HSP27 and HSF1 has been detected in breast cancer biopsies. The striking presence of beta-catenin in the cytoplasm (and not only at the membrane) co-expressed with HSP27 and with HSF1 being also nuclear could have some clinical relevance in terms of prognosis [75]. Notably, HSF1 plays an additional role in the etiology of breast cancer. Unlike normal cells, cancer cells preferentially catabolize glucose by glycolysis, thereby producing high levels of lactic acid. For instance, Zhao *et al.* have shown that ErbB2 promotes glycolysis in a HSF1-dependent manner. Overexpression of ErbB2 increases the expression of HSF1, which binds to *lactate dehydrogenase A* (*LDH-A*) promoter and activates its transcription [76], allowing the metabolization of pyruvate to lactate.

#### 3.1.4. HSF1 and Gastro-Intestinal Cancers

The search for activation of signal transduction pathways in sporadic colorectal cancers (CRC) revealed an increase in the mRNA levels for heat shock and NFκB pathway genes [77]. Namely, an increase of *Hsf1* mRNA in 86% of patients was reported in sporadic colorectal cancer. In that context, HSF1 upregulation in patients was accompanied by an elevated expression of HSP27, HSP90 but also of iNOS (NO synthase; 63%). In addition, HSF1, through the induction of the HSP70 co-chaperone BAG-3, which stabilizes the level of anti-apoptotic Bcl-2 family members, facilitates colon cancer cell survival during pro-apoptotic stress [78]. The oncogenesis of hereditary CRC cancers is believed to involve four signal transduction pathways: (a) the APC-β-catenin-TCF-myc (Wnt) pathway; (b) the microsatellite unstable pathway; (c) the p53 pathway; and (d) the estrogen receptor hypermethylation pathway [79,80]. Although no data are currently available on the potential role of HSF1 in hereditary CRC, at least two of these pathways (Wnt and p53) are known to be affected by HSF1 [77,79,80].

In gastro-intestinal cancer, the level of HSPs is often upregulated with a high variability and without evident correlation with HSF1 level [81]. Instead, the increase observed in HSF1 expression in gastro-intestinal cancer tissues, drives the repression of the pro-apoptotic protein, X-linked inhibitor of apoptosis protein (XIAP)-associated factor-1 (XAF-1) [82]. Thus, HSF1 can impair the apoptotic pathway in cancer cells, at least partly, in a HSP independent manner.

Interestingly, the protein TC1 (a positive regulator of the signaling Wnt/beta-catenin *via* inhibition of Chibby), which is upregulated in an aggressive subtype of gastric cancers, correlates with poor prognosis and has been reported to induce a heat shock response in cancer cells by favoring HSF1 expression. Moreover, HSF1 and TC1 mutually activate each other [83,84].

### 3.2. Role of HSF2 and HSF4 in Glioma and Neuroblastoma

HSP70, HSC70 and HSP90 are found expressed in the tumor parenchyma of all high-grade and most low-grade gliomas, including oligodendrogliomas [85]. A recent work from Mustafa *et al.*, has reported an overexpression of HSF2 (and a modestly elevated level of HSF1) in different stages of glial tumorigenesis, compared to normal brain. The potential involvement of HSF2 in glioma is interesting considering that HSF2 is involved in central nervous system development [5].

Concerning HSF4, while no differences in its expression were found in low-grade glioma and normal brain, it was significantly downregulated in glioblastoma [86].

Neuroblastoma (NB) and Ewing's sarcoma (ES) represent the most common extracranial solid tumors of neuroectodermal origin of childhood. While NB and ES cells globally showed very similar protein expression patterns, GRP78, GRP75, HSC70, HSP70, HSP47, HSP90α, and HSP27 are markedly more expressed in NB cell lines. To date, there is no data related to HSF expression or activity in this type of cancer although it would be worth studying since HSP profile is largely upregulated.

In addition, in human neuroblastoma, the dual-specificity phosphatase 26 (DUSP26) is overexpressed. DUSP26 has been described to inhibit HSF4 phosphorylation induced by Mitogen Anctivated Protein Kinases (MAPK), thereby negatively affecting its ability to bind DNA [87]. Therefore, the negative control exerted by DUSP26 on HSF4, altogether with the inverse correlation in the expression of these two proteins in neuroblastoma, may contribute to repress HSF4 activity in neuroblastoma and its potential crosstalk with HSF1.

### 3.3. Potential Role for HSF3

The expression of HSF3 in the chicken is regulated by the proto-oncogene c-Myb, thus favoring HSP expression, as well as cell proliferation. Moreover, Tanikawa *et al.* reported that a mutated form of p53 is able to inhibit c-Myb mediated cHSF3 transcription allowing tumor progression [88]. Since in humans HSF3 is not expressed, it would be worth studying whether a relation between other HSFs and c-Myb is maintained.

In summary, the principal proteins involved in cancer that have been shown to affect HSF expression or activity are indicated in Table 2.

## 4. HSFs and Cancer Related Targets

**Figure 2.**
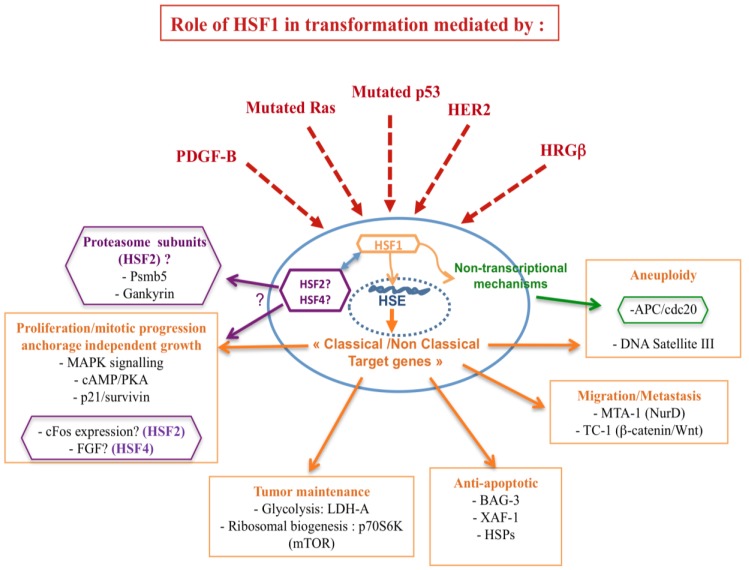
HSF1 potential roles in cancer. Schematic model of the role of HSF1 in cancer transformation: HSF1 activity is hijacked by several oncogenes or mutated tumor suppressors (represented by dashed arrows) allowing activation of a set of genes involved in cancer development, *i.e.*, proliferation and anchorage independent growth, tumor maintenance, anti-apoptotic signaling, migration/metastasis, aneuploidy and probably other mechanisms, including non-transcriptional mechanisms related to the chromatin modification and genomic instability. Notably, there is growing evidence about the role of HSF2 and HSF4 in tumorigenesis since they are directly connected to different cancer related genes (*FGF*, *cFos*, Proteasome subunits). The double arrow represents the crosstalk existing between HSFs.

In light of the involvement of HSFs in mediating tumorigenesis, convincing evidence refers to the impact of HSF1 on the tumor suppressor p53 and the oncogene RAS. Herein, p53 and Ras, pivotal integrators of signaling pathways and key regulators of cell fate decisions, are the two most altered genes in human cancers. Moreover, HSF1 seems to participate to the metastatic potential of the cancer cell through cooperation with different metastatic related genes, such as the prometastatic co-repressor gene MTA1. It is noteworthy that HSF1 possesses additional properties in cancer, such as the enhancement of pro-malignant signaling pathways, involving PKA and TOR [10]. In addition, HSFs act via targets involved in the establishment of genomic instability and epigenetic mechanisms (Figure 2).

### 4.1. HSF1, Tumor Suppressors, and Oncogenes

#### 4.1.1. Tumor Suppressor p53

The first direct *in vivo* evidence of HSF1 implication in the development of spontaneous tumors arises from *p53*^−/−^ mice. The rapid tumor evolution observed in mice lacking p53 mainly results in lymphoma, while *p53*^−/−^*Hsf1*^−/−^ mice rarely develop lymphomas, but rather succumb to other types of cancers, like testicular carcinoma [89]. Tumor suppressor p53 is frequently inactivated by genetic mutations in different cancers [90]. In this context, Dai *et al.* have shown that HSF1 deficiency dramatically reduces spontaneous tumor formation in mice carrying a common, dominant-negative mutation of the *p53* gene, whereas *Hsf1*^+/+^ and *Hsf1*^+/−^ mice bearing dominant-negative mutation of p*53* develop a broad spectrum of tumor types (sarcomas, lymphoma, carcinomas; [10]). This was confirmed by *Hsf1* knock down, using specific siRNAs, in different mouse and human cell systems [10]. These different results observed in these complementary works [10,89] may be explained by differences in the models used, involving distinct mouse genetic background, and the use of clinically relevant *p53* mutation *versus p53* null strategy.

Dysfunction in the proteasome pathway can lead to many disorders including cancers [91], and proteasome inhibitors are currently used in therapeutical approaches [92]. Recently, Lecomte *et al.* have reported that some proteasome subunits, such as Psmb5 and Gankyrin, were significantly downregulated in *Hsf2*^−/−^ MEFs [6]. Interestingly, Gankyrin is an oncoprotein involved in p53 degradation, thus suggesting a potential role of HSF2 in the regulation of the tumor suppressor p53.

#### 4.1.2. Oncogene RAS and HSF1

Compared with wild-type MEFs, *Hsf1*^−/−^ MEFs are refractory to proliferation and resistant to focus formation, driven by oncogenic H-RASV12D or by the proto-oncogene PDGF-B, which are both mitogenic signal transducers [10]. In contrast, expression of c-MYC and LTA (regulator of cell-cycle progression, which are not expected to increase proliferation in already-immortalized cells, but predisposing cells to apoptosis) does not induce proliferation in immortalized *Hsf1*^+/+^ MEFs. Accordingly, *Hsf1*^−/−^ MEFs show no enhanced survival in response to RAS and PDGF/B expression, but reduced survival in response to c-MYC and LTA expression [10].

#### 4.1.3. Oncogene HER2 and HSF1

HSF1 is also required for cell transformation and tumorigenesis induced by the oncogene HER2 (Human Epidermal growth factor Receptor-2), which is responsible for aggressive breast tumors. Indeed, knockdown of *Hsf1* prevents neoplastic transformation (foci formation or tumor growth in xenografts) induced by HER2 expression in untransformed human mammary epithelial MCF-10A cells. This suggests that the proliferation of HER2-expressing cells is critically dependent on HSF1 [63]. Strikingly, this anti-tumorigenic effect of HSF1 downregulation was associated with HER2-induced upregulation of the cyclin-dependent kinase inhibitor p21, a major regulator of senescence in cancer cells [93], and accompanied by a decrease in the mitotic regulator survivin, which is also involved in growth arrest and senescence. Survivin is a member of the inhibitors of apoptosis (IAPs) family and a critical regulator of mitosis by modulation of aurora B kinase [94]. Its decrease is partially controlled by both HSF1 and p21 [63]. In addition, HSF1-mediated control of breast cancer cell senescence was due, at least partly, to the regulation of HSP70 and HSP27 expression.

#### 4.1.4. Loss of Hormonal Control, Epigenetic and Metastasis-Related Targets of HSF1

The potential role of HSF1 in cancer metastasis was first reported in prostate cancer where HSF1 overexpression was correlated with aggressiveness of the tumors [70,71]. Since then, studies have shown that HSF1 is able to mediate tumorigenic effects through the recruitment of the prometastatic co-repressor gene MTA1 (a component of the NuRD complex containing the histone deacetylases HDAC1 and HDAC2) [95]. MTA1 is expressed in numerous human cancers, including breast, prostate and gastro-intestinal cancers and its expression correlates with tumor aggressiveness and metastasis [96–98]. Herein, MTA1 protein expression is higher in hormone-refractory metastatic prostate cancer compared to clinically localized disease and benign prostatic tissues [97].

Breast cancer cell can also escape from hormonal control, leading to higher metastatic potential. Khaleque *et al.*, have reported that HSF1 binds to the corepressor MTA1 in human cultured breast cancer cells and carcinoma samples. The HSF1-MTA1 complex, strongly induced by the transforming ligand heregulin (a ligand for erbB receptor from the EGF family), assembles on the chromatin of breast cancer cells and mainly binds to the promoter of estrogen responsive genes [95]. Thus, HSF1 induces repression of estrogen-dependent gene transcription, an effect linked to cancer invasiveness.

Migration properties in correlation with metastasis potential were also shown to be controlled by HSF1. Mouse embryonic cells derived from *Hsf1*^−/−^ mice are deficient in both basal and EGF-mediated migration. This default in migration, which is partly due to the downregulation of EGF receptor and likely involves Ras, is associated with an impairment of the MAPK signaling pathway [65].

#### 4.1.5. Cdc20/APC Genomic Instability and HSF1

As described above, HSFs have been described to modulate the structure of chromatin and to establish some epigenetic marks (see introduction). In particular HSF1, in association with HSF2, was shown to steer the transcription of the human DNA satellite III in the centromeric regions, establishing an epigenetic status resembling euchromatin [14,99,100]. The involvement of HSF2 in the expression of satellite III DNA was further confirmed [27]. Remarkably, transcription of satellite III DNA is associated with aneuploidy in several cancers such as ovarian cancers, melanoma or myeloma [101–103] and thus could be related to HSF status.

Accordingly, Lee *et al.* have recently shown that HSF1, via its regulatory domain, when overexpressed in radiation induced-fibrosarcoma cells, interacts with cdc20, the co-activator of anaphase-promoting complex (APC), and thus, inhibits the mitotic exit and the ubiquitination activity of APC on two key anaphase inhibitors, Cyclin B1 and securin. As a consequence, this non-transcriptional HSF1 effect leads to aneuploidy and multinucleated cells associated with micronuclei and genomic alteration [104]. In prostate carcinoma cells, a dominant negative construct of HSF1 dramatically alters the DNA content of PC3 cells and inhibits aneuploidy and Cyclin B1 stabilization [73]. Further, HSF1-mediated aneuploidy was facilitated in p53-defective cells. This phenomenon was associated to an increase in HSF1 activity through phosphorylation by a Polo-like kinase, as well as cdc20/HSF1 interaction [105].

Altogether, HSF1 activity is hijacked in a pleiotropic manner by a large diversity of cancer cells and oncogenes to favor tumor initiation and progression. In sharp contrast, it is noteworthy that HSF1 knockout has a minimal effect on the proliferation/survival of normal primary human cells.

### 4.2. Potential Tumorigenic Functions for HSF2 and HSF4

In contrast to HSF1, there is little direct evidence on the involvement of HSF2 and HSF4 in tumorigenesis [106]. Although they can play a role indirectly by their ability to modulate HSF1, several works pointed out their regulatory role in cancer related genes. During mitosis, HSF2 has been shown to bind and activate, not only the HSE promoter of the different *Hsps* (bookmarking), but also the promoter of the proto-oncogene c-Fos. The protein c-Fos is often up-regulated in tumor cells and is well-known for its oncogenic activity [107]. Thereby, it is tempting to conclude that HSF2 might regulate the oncogenic potential of c-Fos [108].

In addition, HSF2 is a labile protein whose degradation was recently shown to be regulated by anaphase-promoting complex/cyclosome (APC/C), a ubiquitin E3 ligase, which drives the degradation of cell cycle regulators in cycling cells by associating with the coactivators Cdc20 and Cdh1 [28]. Therefore, APC/C, by its effect on HSF2, could be involved in a cell cycle-dependent manner in the modulation of HSF1 activity, which could have profound effects on cancer progression.

Finally, the oncomiR miR18 has been shown to regulate the expression of HSF2 during spermatogenesis [52]. It could be possible that miR18 or other miRNA could regulate HSF2 expression in cancer cells, which, by modulating HSF1, could influence cancer initiation or progression.

HSF4 regulates normal cell proliferation and differentiation during mouse lens development, and belongs to the first group in the hierarchy of genes involved in this process [5,59]. Interestingly, HSF4, through its effect repressing the expression of the *Fibroblast Growth factor-7* (*FGF-7*) gene which encodes a specific mitogen for epithelial cells, is also important for governing ocular surface morphogenesis. Upregulation of FGF-7 has been reported to be associated with many human neoplastic tumors of epithelial origin including pancreatic, breast and gastric cancer, thus underlying the oncogenic role of FGF-7 [109–111]. Most importantly, Chimaka *et al.*, have demonstrated that overexpression of FGF-7 in Keratin 12-rtTA/tet-O-FGF-7 double transgenic mouse model, in which FGF-7 expression in corneal epithelium is driven by doxycycline treatment, induces epithelial hyperplasia that mimic the human ocular surface squamous neoplasia (OSSN) [112]. In light of this work and the observation that FGF-7 is upregulated in *Hsf4*^−/−^ lens epithelial cells, a likely scenario is that HSF4 may interfere with the tumorigenic function of FGF-7 [4].

## 5. The Inhibition of HSFs in Cancer Therapy

### 5.1. Targeting HSF1

HSF1 knockdown experiments in cancer cells demonstrate the interest of blocking this transcription factor in cancer therapy. A study using different human HSF1 targeted shRNAi constructs was performed using a collection of breast cancer cell lines that differed in their p53 status, HER2 expression, estrogen sensitivity and metastatic potential. All cancer cells were strongly affected by the HSF1–inhibitory hairpins [10], Further, Rossi *et al.*, have determined that the ideal size target for siRNA mediated HSF1 silencing is 322–340 nucleotides. By generating a pSUPER-HSF1 vector, able to potently suppress HSF1 gene, they dramatically increased the sensitivity to hyperthermochemotherapy (combination of a Cisplatin treatment with heat-shock), leading to massive apoptosis of Hela cervical cancer cells [113]. These approaches, albeit attractive, are still not established for clinical use. In this respect, an expanding array of small, drug-like compounds is currently available, some with potent HSF1-modulating activity in organisms, but only a few of them are in clinical trials (see below).

### 5.2. Chemical Inhibitors of HSF1

Chemical inhibitors of HSF1 activation have been described including quercetin, genistein and the synthetic benzylidene lactams, KNK437. The natural product Stresgenin B has also been reported to inhibit induction of HSPs, but its mode of action is still unknown [114]. Quercetin increases the sensitivity of drug-resistant cancer cells to anti-cancer agents [115], and sensitizes cancer cells to hyperthermia, cisplatin [116] and tiazofurin [117]. HSF1 is strongly depleted following quercetin treatment and this effect appeared more marked in neuroblastoma cells. Moreover, a strict correspondence between the quercetin concentrations necessary to cause both HSP inhibition and doxorubicin sensitizing effect was observed, suggesting that neuroblastoma cell' resistance to doxorubicin treatment might be due to high levels of HSPs, under the control of HSF1. As for quercetin treatment, neuroblastoma cells were the most sensitive to HSF1 silencing effect. Knockdown of HSF1 in these cells strongly increased the anti-proliferative and pro-apoptotic effects induced by cisplatin [118]. Although efficient in cancer experimental models, the clinical use of these inhibitors is limited because of their pleiotropic effects [119].

To date, the most potent inhibitor of the HSF1 transcriptional function is Triptolide, a diterpene triepoxide from the plant Triptergium wilfordii. Triptolide does not interfere with the early events leading to trimer formation, hyperphosphorylation and DNA binding of HSF1 [120]. Albeit toxic at high concentrations, a therapeutic dose of Triptolide has been defined to treat pancreatic cancer xenografts [121]. Nevertheless, the precise mechanism of triptolide action remains to be investigated.

Several cancer treatment approaches, such as HSP90 inhibitors (geldanamycin) and proteasome inhibitors (Bortezomib), induced a proteotoxic stress that activates a pro-survival pathway, which explains the low efficiency of these therapies [122]. Both HSP90 and proteasome inhibition are known to activate HSF1 and HSF2 [21,25,123–125]. From a high-throughput screening of molecular products that can interfere with the heat shock response, two new compounds were identified namely, NZ28 and emunin (emetine derivative). These two molecules exhibit little acute toxicity and allowed a strong sensitization of myeloma cells to proteasome and HSP90 inhibitors, as well as of prostate carcinoma cells to proteasome inhibitors [126]. Their molecular action is not yet known but seems to involve post-transcriptional events downstream HSF1. Similarly, by a high content target based screening, Au *et al.*, have identified another small molecule inhibitor of HSF1 transcriptional activity [127]. This molecule substantially hampers granules formation in heat-shocked Hela cells and significantly inhibits HSF1 phosphorylation. This effect is associated with a reduction in HSP70 and HSP90 expression. Recently, a malaria drug, quinacrine (QC) has been reported to prevent the induction of HSF1-dependent transcription of *hsp70* gene in a relative selective manner. The combinational treatment with a HSP90 inhibitor (17-DMAG) results in suppression of tumor growth in mouse syngeneic models [122].

### 5.3. Viral Approaches

Recently, an interesting anti-cancer approach has been developed based on the fact that HSF1 overexpression enhances the oncolytic effect of a replicative adenovirus. E1B55kD deleted oncolityc adenovirus, Adel55, was designed to achieve cancer specific cytotoxicity. A construct bearing Adel55-cHSF1 for tumor gene therapy, demonstrated that it favored oncolysis and viral replication by increasing its burst in breast cancer cells and SW620 xenografts [128]. The future will tell of the feasibility of such a viral therapy in cancer patients.

### 5.4. Targeting other HSFs

It has recently been reported that, in *Hsf2* knockout cells, the proteasome activity is lower compared to normal counterparts. This inhibitory effect is due to the regulatory role of HSF2 on the transcription of some proteasome subunits. Therapeutic use of proteasome inhibitors participates to the selection of the resistant clones, which are associated to the induction of beta5 and beta2 proteasome subunits expression [129,130]. Since in *Hsf2*^−/−^ MEFs one of these two subunits is downregulated, this suggests that inhibition of HSF2 could be beneficial for the sensitization of cancer cells to proteasome inhibitors. More importantly, HSF2 regulates Gankyrin expression that is responsible for p53 tumor suppressor degradation [6]. Targeting HSF2 could stabilize p53 and favor apoptosis. Therefore, these observations suggest that specific HSF2 inhibitors could reduce the chemoresistance to proteasome inhibition that is so frequently observed in clinical studies. This pioneer study defines a new cancer research axe focused on HSF2 that definitely deserves further investigation.

## 6. Concluding Remarks

HSF1 expression is likely to be crucial for the “non-oncogene addiction” and the stressed phenotype of cancer cells. These stresses include proteotoxic and oxidative stress, frequent spontaneous DNA damage and aneuploidy [69]. HSF1 by itself does not act as a classical oncogene or tumor suppressor. Neither enforced overexpression nor knockout directly drives transformation. HSF1 acts as a major multifaceted enhancer of tumorigenesis by regulating diverse core cellular functions that include proliferation, survival, protein synthesis and glucose metabolism, and therefore, is an attractive potential target in cancer therapy. However, some considerations must be taken when using HSF1 inhibitors because, although they seem beneficial in cancer treatment, they might, in parallel, accelerate neurodegenerative processes and aging. Indeed, HSF1, partly through the induction of chaperones that inhibit protein aggregation like HSP27, displays a protective effect against neurodegenerative diseases [131,132]. In this respect, therapeutic induction of HSF1-mediated stress response by non-toxic agents, like HSP90 inhibitors and Celastroloids, is currently being explored in Huntington disease [131,132]. It should also be mentioned here that hyperthermia, a strategy in cancer that could be considered as the opposite of HSF inhibition, has proved its therapeutical value in clinical trials and is still used in association with chemotherapeutic drugs in some countries (Germany, Italy, Japan, [133]). Therefore, regarding the development of new tools/potential drugs targeting HSFs, it seems important that compounds do not cross the blood-brain barrier in order to minimize neurodegenerative risks and that they take into account HSF3 multiple post-translational modifications, as well as the formation of heterocomplexes between them. Ideally, rather than to simply activate or inhibit a given HSF, it would be more relevant/innovative to target a specific regulatory status, allowing a restrictive effect on designated signaling pathways.

## Figures and Tables

**Table 1.** HSF main characteristics.

	**HSF1**	**HSF2**	**HSF3**	**HSF4**
**Species Conservation**	Human, Mouse, Chicken	Human, Mouse, Chicken	Chicken, Mouse	Human, Mouse, Chicken
**Oligomerization**	NC: Monomer HS: Trimer	HS: Dimer-heterotrimers HSF1/HSF2 Differentiation: Dimer-heterotrimers HSF1/HSF2 Development: homotrimers/heterotrimers HSF1/HSF2	NC and HS: Dimer	NC and HS: Trimer
**Target genes in stress responses**	*Hsp* genes (+ non-classical stress genes)	*Hsp* genes (+ non-classical stress genes) Proteasome subunits	Mainly *Hsp* genes in chicken Non classical stress genes in mice	Not heat-responsive Repression of hsp genes in ectopic expression experiments
**Developmental role of HSF**	Oogenesis, maternal factor, development and maintenance of germ, ciliated and immune cells	Brain and gametogenesis development	ND	Development and maintenance of sensory organs
**Adult tissue localization**	Ubiquitous	Ubiquitous	Ubiquitous	Tissue specific (heart, brain, skeletal muscle, and pancreas)

NC: Normal conditions; HS: Heat shock; ND: Not determined.

**Table 2.** HSF regulation in cancer.

**HSF**	**HSF Positive/Negative Regulators of interest in cancer**	**Effect on HSFs**
HSF1	HRGβ	Increase HSF1 expression => activation of LDH-A (glycolytic enzyme) and the formation of the complex HSF1/MTA1 (prometastasic)
HSF1	TC1 (Wnt Signaling)	Increases HSF1 expression in gastro-intestinal cancer => tumor aggressiveness
HSF1	GSK3	Inhibits HSF1 activity-GSK3 is inhibited in breast cancer
HSF2	OncomiR18	Inhibits HSF2 expression in spermatogenesis
HSF4	DUSP26	Inhibits HSF4 activation induced by MAPK
HSF3	c-Myb	Induces HSF3 expression
